# Gradient metapopulation microfluidic ecologies shape genetic and biofilm drivers of T4r phage resistance in *E. coli*

**DOI:** 10.1038/s41522-026-00959-z

**Published:** 2026-04-25

**Authors:** Krisztina Nagy, Sarshad Koderi Valappil, Trung V. Phan, Shengkai Li, Laszlo Der, Ryan Morris, Julia Bos, Sophia Winslow, Peter Galajda, Gabor Rakhely, Robert H. Austin

**Affiliations:** 1 Institute of Biophysics, HUN-REN Biological Research Centre, Szeged, Hungary; 2 Department of Experimental Physics, Institute of Physics, University of Szeged, Szeged, Hungary; 3 Department of Biotechnology, University of Szeged, Szeged, Hungary; 4 Environmental Genomics and Systems Biology Division, Lawrence Berkeley National Laboratory, Berkeley, CA USA; 5 Department of Chemical and Biomolecular Engineering, Johns Hopkins University, Baltimore, MD USA; 6 Department of Physics, Princeton University, Princeton, NJ USA; 7 School of Physics & Astronomy, University of Edinburgh, Edinburgh, UK; 8 Institut Pasteur, Université Paris Cité, CNRS UMR 3525, Unité Plasticité du Génome Bactérien, Paris, France; 9 University of Northwestern St. Paul, Roseville, MN USA

**Keywords:** Molecular evolution, Biofilms

## Abstract

We use a gradient microfluidic metapopulation ecology which generates non-uniform phage concentration gradients and micro-ecological niches to reveal the importance of time, spatial population structure and collective population dynamics in the de novo evolution of T4r bacteriophage resistant motile *E. coli*. An insensitive bacterial population against T4r phage occurs within 20 hours in small interconnected population niches created by a gradient of phage virions, driven by evolution in transient biofilm patches. Sequencing of the resistant bacteria reveals mutations at the receptor site of bacteriophage T4r as expected but also in genes associated with biofilm formation and surface adhesion, supporting the hypothesis that evolution within transient biofilms drives de novo phage resistance.

## Introduction

Bacteriophages (phages) are viruses that infect bacteria. Phages coexist with microbes, playing a fundamental role in microbial diversity, population dynamics, and evolution. Understanding the interaction between phages and bacteria gives us fundamental information on ecological and evolutionary processes^1^. Phages can be divided into two classes: temperate and virulent. The life history for temperate phages typically consists of two parts: a lysogenic phase after inserting their DNA into the genome of a host bacterium after infection, and a propagating lytic phase where they extract their genome from the host genome, reproduce within the bacterium, and exit by lysis of the bacterium. Virulent phages only have a lytic cycle; they do not insert their DNA into the host but rather use the cell’s machinery to make copies and lyse the cell.

Bacteriophage T4 can pause cell lysis in super-infected bacteria to yield very high titer yields in the lysis of remaining bacteria. The T4 phage strain used in this study is highly virulent and rapidly lyses the cell after infection (it is an obligate lytic^2^). Phage T4r (T4 rapid) lacks the lysis inhibition (LIN) genes of wild-type T4 and rapidly lyses bacteria, even multiply infected “super-infected” bacteria, with resultant relatively low titer yields compared to wild-type T4^3^. Because of the rapid lysis of T4r infected bacteria any survival of a low number *N*_*O*_ of bacteria can only be due to either previously acquired resistance (random mutations) or a kind of rapid response by the stress-challenged bacteria.

The well-known studies of Luria and Delbrück in the 1940s, carried out with the obligate lytic phage T1 and *E. coli*, proved that random mutations conferring resistance occur in bacteria regardless of the presence of selective pressure. Since then, we have learned that mutations in response to stress also occur^4^ and that there are different ways for organisms from bacteria to man to acquire heritable phenotypic changes upon transient exposure to stress^5,6^. We also know that both genome-wide mutation rate and the mutation rates of specific genes can be affected by the environment^7–9^. Furthermore, the complexity of the environment (e.g., compartmentalization) might facilitate the fixation of mutations in bacterial populations^10,11^. In the past decade more and more papers highlighted the importance of spatial variations in antibiotic concentration gradients in the evolution of bacterial antibiotic resistance^12–15^. We have shown in a previous publication that stress gradients imposed over a metapopulation of weakly coupled communities can greatly increase the rate at which resistance evolved to the mutagenic antibiotic ciprofloxacin^14^.

In this study, we examine how stress gradients have an impact on the emergence of phage resistance in small interconnected *E. coli* populations with time. Bacteria can develop resistance de novo against phage infections in different ways: by genetic mutations and by an “adaptive immune response” using the CRISPR-Cas system to detect and destroy DNA from similar viruses during subsequent infection^16,17^. Laboratory strains of *E. coli* possess a functional CRISPR-Cas system, however, this system seems to be “silent” under normal laboratory conditions^18,19^. Although *E. coli* is an important model organism for the characterization of the CRISPR-Cas systems, and detailed descriptions of its structure and molecular mechanism in this species can be found in the literature^20–22^, the physiological conditions needed for its activation is still unclear and it needs to be further investigated.

For a realistic bacterial-phage scenario it is important to try and replicate the natural habitats of microorganisms, which are complex: the environments have physical heterogeneity and heterogeneous distribution of resources. However, most studies have been carried out in well-mixed populations or chemostats^23^ or on agar plates with homogeneous phage concentrations. These studies do not capture some important complexities of natural communities (e.g., spatial^24^ and temporal^25^ heterogeneity). Microfluidics provide excellent tools to mimic such aspects of natural habitats and study questions related to microbial ecology^26^. For example, a microfluidic mother machine study revealed the importance of the presence of spatial refugees in an *E. coli* population against bacteriophage T4 in structured environments compared to well-mixed cultures^27^. They suggest that structured environments promote the selection of phenotypic variants with low phage receptor expression. Other studies suggest that the opportunity of cell aggregation and biofilm formation provided by spatially structured environments could lead to different types of interactions and evolutionary pathways as opposed to shaken liquid cultures in test tubes^28–30^.

In this paper, we aim to shed light on the fundamental processes behind the evolution of bacteriophage resistance. We use a microfabricated environment in which we exposed motile *E. coli* bacteria to spatial gradients of bacteriophage T4r (The “r” in T4r denotes a mutation that leads to rapid lysis of the bacterial host) which reproduce by the lytic cycle when infecting *E. coli*. The growth and distribution of the bacterial population were followed in time by fluorescence time-lapse microscopy. Resistant cells were collected from the microfluidic device for further analysis, e.g., genomic sequencing was performed to identify key mutations leading to the observed resistance.

## Results

### Colonization of the microstructured habitat by motile bacteria in the presence of phage gradient

**Fig. 1:**
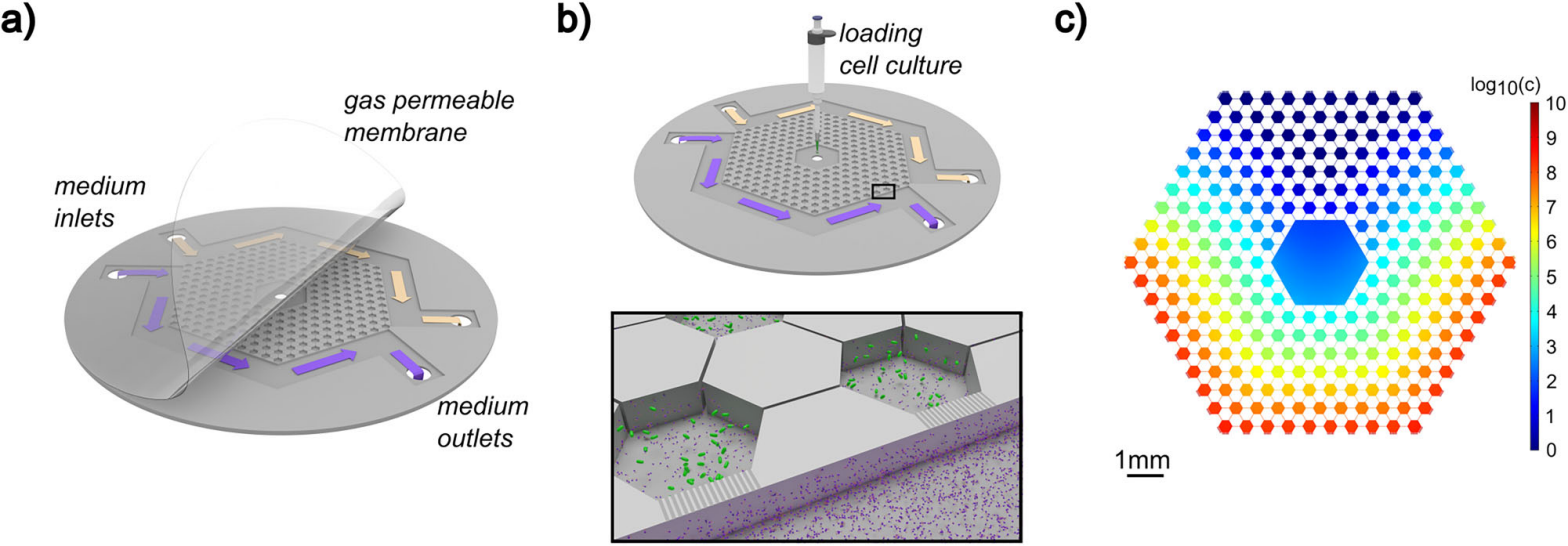
The microfluidic setup. **a** 3D drawing of the microfluidic device (not-to-scale). The etched silicon chip is sealed with a 25 µm thick gas permeable LUMOX film, pressurized from the front. Arrows indicate the direction of medium flow. Yellow color is used for pure LB (top channel) and the purple color corresponds to LB supplemented with T4r phages (bottom channel). **b** Mounting of the chip in a LUMOX dish with applied external sealing back air pressure. Bacteria are inoculated into the middle inlet hole with a pipette. The inset shows the phage (purple particles) gradient forming from the bottom channel. Shallow (100 nm deep) nanoslits connect the side channels and the outer hexagon chambers. **c** Simulation of the phage gradient present in the device at the initial stage of the experiment. Phage concentration *c* is indicated by the colorbar in logarithmic scale. The unit of c is virion/ml.

A microfabricated landscape was used to study the evolution of bacteria against lytic phages. The microfluidic device consisted of a network of hexagon microchambers that were connected through narrow corridors (see “Methods”). Nutrient supply was ensured by a constant flow of fresh LB medium in the side channels that were connected (by nanoslits) to the outer patches (Fig. 1a, b). Motile *E. coli* cells were inoculated into the inlet hole positioned at the center of the device and can move between the microchambers and find the most favorable conditions. The dimensions of the nanoslits (100 nm x 60 µm x 7 µm) prevent the escape of cells from the array, however, it allows the free diffusion of chemicals and bacteriophages into the system. The initial size of the bacterial population loaded into the center was 10^4^ cells. In the absence of any phage gradients, bacteria form chemotactic waves^31^ and move over to the nanoslits which act as nutrient sources in this system. This rapid chemotaxis (which takes a couple of hours) towards the nanoslits occurs because of the extremely small volume (approximately 0.4 *µ*l) of medium within the hexagon array, and the rapid local consumption of nutrients by bacteria.

As a first step, before each experiment, we set up an initial T4r phage gradient across the hexagon array, thus the inoculated bacteria were immediately in contact with lytic T4r phage. The initial distribution of phages in the microfluidic device, in the case of flowing LB medium supplemented with phages (2 × 10^9^ virus particles/ml concentration) in the bottom side channel, was estimated by a 3D model and is presented in Fig. 1c. Based on our simulations, this is not a steady state, the gradient slowly changes throughout the experiment. Also, the lysis of bacteria infected by phage disturbs the phage gradient in the later phase of the experiments. T4r, lacking the LIN genes^3^, causes rapid lysis of *E. coli* with a relatively low titer yield compared to wild-type T4 phage, however, infected cells might still disturb the gradient in the system. The local increment of phage concentration upon lysis events is not included in the 3D model, but it represents well the initial microenvironment.

In the case of T4r phage gradient the previously mentioned fast chemotactic waves were lacking. Instead, localized emergence of insensitive sub-populations at low-intermediate phage concentrations was the typical response we observed. We call the locations, where the insensitive population starts intense growth and colonization, “hot-spots”. These hot-spots appeared within 12–36 h. The exact location and timing of the response was characterized by calculating the average occupancy of the microchambers based on the fluorescence images taken during the experiment. Based on the occupancy data, we can say that there is no specific location within the device for the emergence of insensitive populations, however, it never happens on the high phage concentration side of the hexagon array (Supplementary Fig. 1). The population starts to grow in the central regions (at moderate phage concentration levels), and spread from there. This observation suggests that the initial bacteriophage gradient influences the population dynamics at least to some extent.

**Fig. 2:**
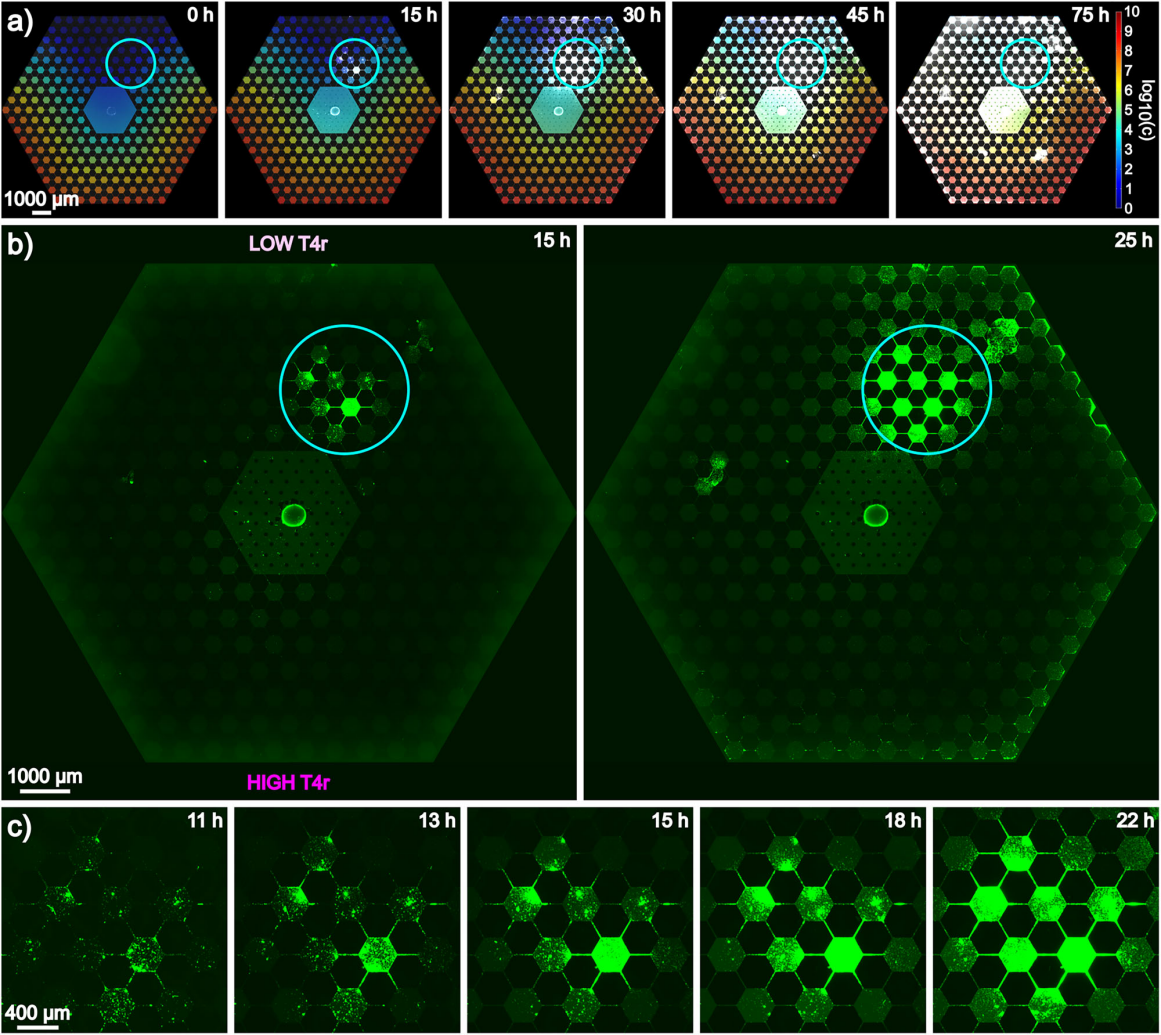
The emergence of insensitive bacterial population in T4r gradient. **a** Snapshots of fluorescence microscopy images overlaid on the calculated T4r concentration gradient over a period of 75 h. The emergence of an insensitive population at the low-phage concentration side of the device is outlined by a solid circle. Phage concentration (**c**) is represented in logarithmic scale. The unit of c is virion/ml. **b** Stitched image of the full microfluidic array from 15 to 25 h after inoculation. The solid blue circle outlines the hot spot of the insensitive bacterial population against T4r phage. **c** Emergence of insensitive sub-population from small clusters of bacteria over a time scale from 11 to 22 h.

Figure 2 presents a representative image series on the basic progression of motile *E. coli* in the device. Figure 2a shows fluorescence snapshots overlaid on top of the calculated concentration profile over the 75-h-long experiment. Supplementary Movie 1 and 2 contain the entire time-lapse image series of the experiment. The “hot-spot” is outlined by the solid circle in Fig. 2a, b, whereas Fig. 2c shows zoom-in images of this region. We can see aggregation of cells and the formation of small clusters from which bacteria spread to other parts of the device as well (Fig. 2b, c). Note, that in Fig. 2c it can be seen that after 25 h single cells also appear at the nanoslits even at the high phage concentration side of the hexagon array.

**Fig. 3:**
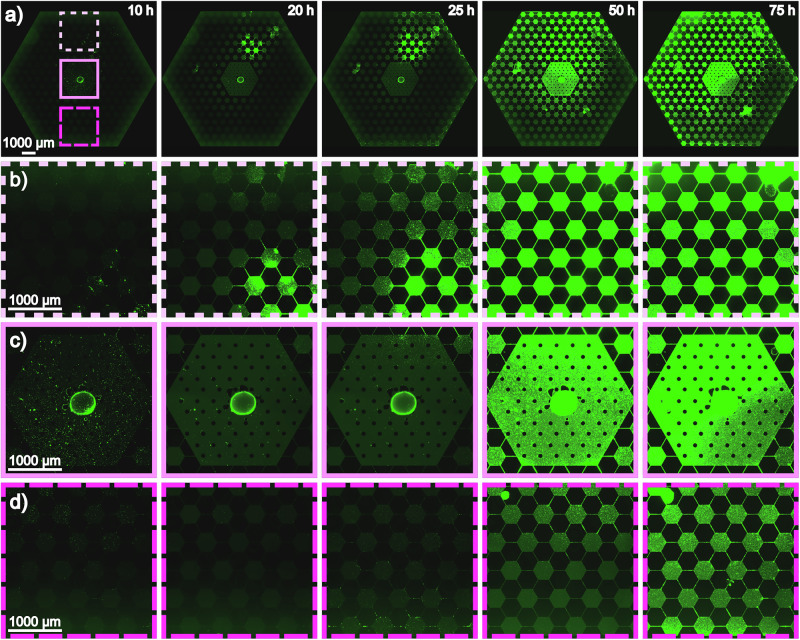
Progression of bacterial growth within the whole microfluidic stress landscape. **a** Stitched images of the full array from 10 to 75 h after inoculation. The dashed and solid squares outline regions of the habitat with different phage concentrations. **b** Zoom-in images of the top region of low T4r density. **c** Zoom-in images at the inoculation region in the center of the device. **d** Zoom-in images of the bottom region of high T4r density.

The observed progression pattern is the result of different mechanisms: (1) chemotaxis towards the nutrient sources, (2) response to the stress exposed by phage. The latter one might imply e.g., producing extracellular materials, biofilm formation or the appearance of stress-induced mutations in the complex environment. The details of the progression is presented in the fluorescence images of Fig. 3. by zooming into the different regions (with different phage concentrations) of the microarray habitat over the time scale of an experiment (same as presented in Fig. 2.). Figure 3. shows that after 10 h there are bacteria in the outer hexagon chambers (right next to the nanoslits). Bacteria spread fast on the phage-free/low-phage concentration side of the habitat. However, cells (mostly single cells) can be detected on the high phage concentration side as well within 10 h. Figure 3a and Supplementary Movie 1 shows that 48 h is enough for the population to colonize the whole habitat. However, dark areas can be clearly identified in the landscape (Fig. 3a) that probably represent regions of lyzed cells as a consequence of local phage infections. Occasionally, bacteria form such dense biofilm-like structures that slightly lift the sealing membrane. This was not observed before when using the same setup in evolution studies against antibiotics^14^.

### Characterization of bacteria extracted from the microarray habitat

Three biological replicates were performed and the dynamics of the bacterial populations in the bacteriophage stress landscape were followed over 3–4 days. In all cases, the insensitive sub-populations appeared within 36 h (Supplementary Fig. 1) and the basic progression pattern was very similar to what is described and presented in Fig. 3. After each experiment, the device was taken apart to collect cells. For this purpose, the Lumox cover of the ecology was removed from the silicon and resistant clones were isolated using selection plates (see “Methods”). Three colonies were randomly selected for further analysis. The growth properties and whole-genome sequencing data were compared to the ancestor *E. coli* strain (see Methods).

The results of whole-genome sequencing confirmed the presence of mutations related to phage resistance in the cells that were exposed to the T4r phage gradient during the experiments. The relevant mutations are summarized in Table 1.

**Table 1:** Summary of the relevant mutations found in resistant mutants extracted from the microfluidic device after exposure to a gradient of T4r

Sample	Gene	Description	Mutation	Position
mutant1	*ompC*	outer membrane protein C	MC (8 bp)	201,809
	*csgB*	minor curli subunit	MC (526 bp)	1807–2333
	*csgD*	CsgBAC operon	MC (155 bp)	7607–7761
mutant2	*csgB*	minor curli subunit	MC (482 bp)	1851–2333
	*csgD*	CsgBAC operon	MC (155 bp)	7607–7761
	*rfaP*	outer membrane lipopolysaccharide core	MC (218 bp)	85622–85839
	*rcsC*	sensor histidine kinase RcsC	T → G (CTG → CGG)	196,992
mutant3	*ompC*	outer membrane protein C	+T (TAT → TTT)	201,731
	*rfaP*	outer membrane lipopolysaccharide core	MC (218 bp)	85622–85839

The most obvious mutations related to T4r resistance occurred at the receptor site of bacteriophage T4 (*ompC*)^32–34^. Two out of the examined three isolates (mutant1 and mutant3 in Table 1) had changes in this particular gene, which could serve as a primary defense mechanism by inhibiting phage adsorption. The other detected mutations could also contribute to the reduced entry of the phage into the cell, and most of them can be associated with the adhesion properties and biofilm-forming abilities of *E. coli*. E.g., the rfa locus is important in the barrier function of the outer membrane. *rfaP* is involved in the pathway of the LPS core biosynthesis^35^. Besides *rfaP*, mutations were detected in *csgB*, *csgD*, and *rcsC*, which are important in the normal biofilm formation of *E. coli*^36,37^. In two samples (mutant1 and mutant2) both *csgB* and *csgD* were altered. These genes have a crucial role in the expression of curli fimbrae and possess a positive role in biofilm formation^30^. *rcsC* is associated to mucoid phenotype and to normal biofilm formation on solid surfaces^38^. It is also involved in colanic acid synthesis. Indeed, mutant2 having an SNP in *rcsC*, has a mucoid phenotype when forming colonies on a hard agar surface. The sequencing results together with the time-lapse images (Fig. 2, 3) suggest that biofilm formation has a crucial role in bacterial phage resistance.

In the past decade, the role of clustered regulatory interspaced short palindromic repeats (CRISPR) and the associated *cas* genes in resistance against phages got acknowledged^16^. The activity of the CRISPR system of *E. coli* had not been reported previously under normal laboratory conditions. However, we were curious if the structured landscape we designed could somehow induce it. For this purpose, we compared the CRISPR regions of the isolates to the ancestor strain. No changes could be detected in the spacer sequences.

**Fig. 4:**
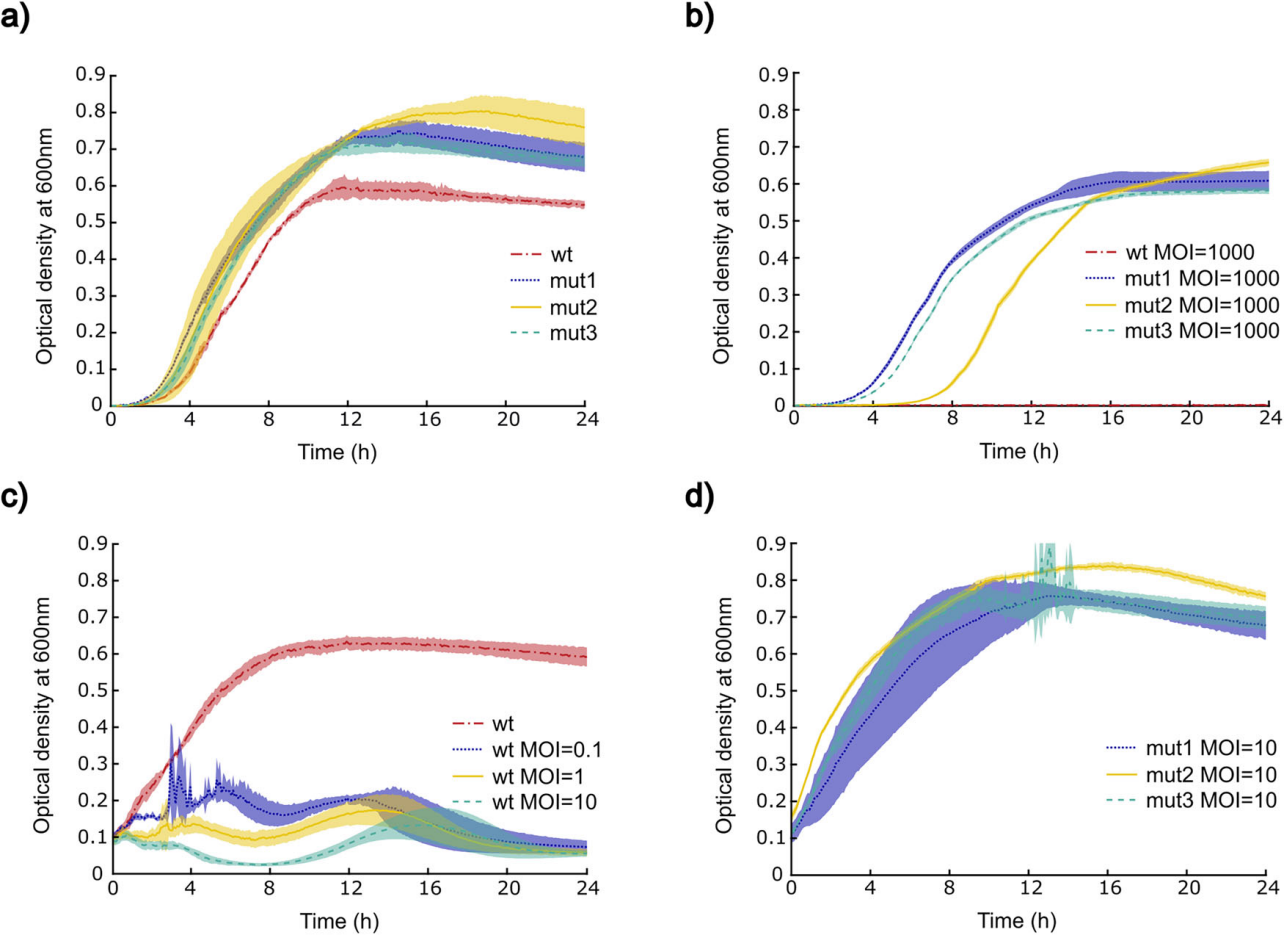
Growth properties of the ancestor and three mutant *E. coli* strains. **a** Growth of the ancestor (red dashed line) and three mutant (mut1: dotted blue line; mut2: continuous yellow line; mut3: dashed green line) strains in phage-free LB media. **b** Growth of the ancestor (red dashed line) and 3 mutant (mut1: dotted blue line; mut2: continuous yellow line; mut3: dashed green line) strains in the presence of high phage concentration (MOI = 1000, the initial cell number is 100,000). **c** Growth of the ancestor strain in LB media at different MOI. T4r was added to mid-log phase bacteria culture, the initial bacteria cell number is 10,000,000. **d** Growth of the three mutant strains when applying phage (MOI = 10) at mid-log phase culture. The initial cell number is 10,000,000 (mut1: dotted blue line; mut2: continuous yellow line; mut3: dashed green line).

The growth properties of the mutants in the presence and absence of T4r were compared to the ancestor strain by measuring the optical density of cell cultures in 96-well plates (see Methods). Different multiplicity of infection (MOI) scenarios were applied to get a more detailed picture on the bacteria-phage interaction. Figure 4a shows that there was no apparent fitness cost to achieving T4r resistance. The growth curves of the mutants are similar regardless of the presence of the phage (Fig. 4a, b). The high-level resistance against T4r is shown in Fig. 4b, where the bacteria-phage cultures were started by applying MOI = 1000. Under such circumstances, the growth of the ancestor strain was completely excluded. Addition of T4r to a growing culture of the ancestor bacterial strain at mid-log phase at lower MOI values (from 0 to 10) results in no change to the further growth of the resistant strain but the lysis of wild-type bacteria, as shown in Fig. 4c, d.

The presence of mutations in *csgB*, *csgD* and *rcsC* genes, suggest that the mutants show differences in biofilm-forming and surface adhesion properties compared to the ancestor strain. Therefore, we probed the mutant strains for their ability to form surface-adhered biofilms using the crystal violet staining protocol^39^. Interestingly, the mutant strains had substantially reduced biofilm-forming ability (see Supplementary Fig. 2).

**Fig. 5:**
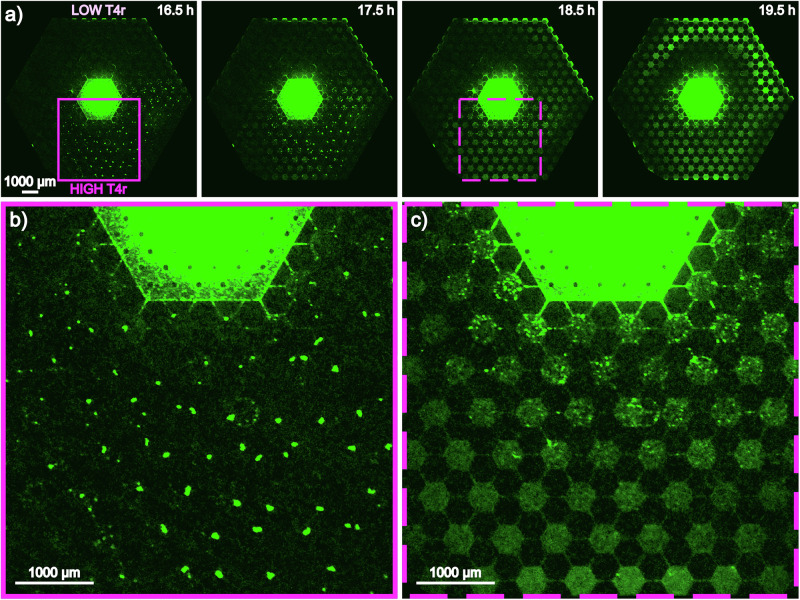
Expansion of resistant *E. coli* in a gradient of T4r phage. **a** Snapshots from 16.5 h after inoculation to 19.5 h. **b** Expanded view at 16.5 h of the region at high T4r concentration where the bacteria transiently form small clusters. **c** Rapid dissolution of the bacterial aggregates and movement to nanoslits.

Inoculation of the resistant bacteria into the same complex ecology (with T4r gradient) that resulted in the evolution of the resistant strain shows a more nuanced picture of the changed growth properties of the mutants than the 96 well-plate experiments of Fig. 4. Figure 5 and supplementary movie 2 shows the rather complex response of the mutant strain (mutant 1) in a T4r gradient and a set of connected habitats. At the low T4r titer side, as expected we see movement to the nanoslits and growth (Fig. 5a). At the intermediate and high titer concentrations, we see a transient aggregation of the bacteria into small clusters within each microhabitat for a period of several hours, followed by the dissolution of the clusters and uniform growth even at high titers of T4r (Fig. 5b, c). The chemotactic waves—that are typical for *E. coli*—were also present under such circumstances (supplementary Movie 3).

## Discussion

Bacteria and phages coexist in nature performing a continuous co-evolutionary co-existence^40–42^. As part of this evolutionary co-existence, bacteria have evolved different mechanisms to survive phage infections and phage have evolved mechanisms not to destroy their hosts, whom they need. The natural environment of the perhaps somewhat one-sided but still symbiotic relation between phage and bacteria^43^ is not the well-stirred chemostat of the microbiology laboratory^44^. Such systems do not capture some important complexities of natural communities. For example, spatial and temporal inhomogeneities in the distribution of stress factors (selection pressure) have a profound impact on evolutionary processes, changing population numbers and the invasion of resistant strains into sensitive ones. Understanding how the environment and ecology can change both mutation rates and selection dynamics is important to properly describe evolutionary processes^45^.

Microfluidics offers state-of-the-art tools to mimic the complexity of natural environments^26,46^. In this study we created a complex stress landscape that consists of an array of microhabitats with different concentrations of the lytic bacteriophage T4r. The microchambers are connected through narrow channels allowing bacteria to move around in the landscape. In this platform we studied the growth and progression of a small population of a fluorescently labeled motile strain of *E. coli*.

In our experiments, insensitive bacterial populations emerged within 12–36 h. There is no specific location within the device where these populations appear. However, they generally appear and spread from the central regions and not at the extreme microenvironments, close to the side-channels, where the phage concentration is the lowest or the highest. These regions are also further away from the nutrient source of the system. This observation is in contrast with what the research group observed previously in case of antibiotic gradients. In the same system, antibiotic resistant *E. coli* populations emerged at locations with the sharpest gradients and the best nutrient supplies^14^. Based on our simple model calculations taking into account the dimensions of the microfluidic device and the diffusion of the bacteriophage (see “Methods”), the observed “hot-spots” belong to low-intermediate initial phage concentrations. Unfortunately, the complexity of the system does not allow us to create a precise mathematical model taking into account the continuously altering size of the bacteria and phage populations and the local concentration changes due to the lysis of infected cells. Nevertheless, repeated emergence of an expanding sub-population in the central region of the device (see the occupancy maps in Supplementary Fig. 1.) suggests the importance of the microenvironment and the initial distribution of phages. Completely random mutations in such a small population would result in resistant sub-populations to pop up at random locations in the device.

Typically, bacterial strategies for survival in complex environments involve both phenotypic and genetic adaptations. In terms of genetic adaptation, ordinarily *E. coli* have a remarkably faithful DNA replication system with an error rate on the order of 10^*−*9^/bp/generation^47^ in the absence of stress. In our previous antibiotic evolution experiments we triggered the highly error-prone DNA replication response of the SOS system in *E. coli*^48^, driven by the appearance of single-stranded DNA due to gyrase A blockage by ciprofloxacin. This led to the formation of filamentous bacteria^49^ and an accelerated evolution^50^.

Responses to phage predation could be very complex and diverse, depending greatly on the bacterial host, phage and the multiplicity of infection. Over the past decades more and more studies highlight the importance of adaptive mutations and the role of environmental factors in the evolution of resistance, leading to a continuity between random (“Darwinian”) and directed (“Lamarckian”) mutations. E.g., stress-induced mutations, activation of mobile elements, horizontal gene transfer can be considered as quasi-Lamarckian phenomena, and in the case of resistance against bacteriophages, the prokaryotic adaptive immunity mediated by CRISPR-Cas systems possesses almost all criteria to be considered Lamarckian^51,52^. Holmes et al.^53^ revisited the original Delbrück-Luria experiments and have carried out an extensive analysis. In there calculations, they have included both spontaneous (Θ_*D*_) and stress response (Θ_*L*_) mutation rates. They created a Composite model which included both mutation mechanisms. Based on their calculations, the pure Lamarckian model is inconsistent with the experimental data (as expected), but the Composite model (containing both mechanisms) and the pure Darwinian model fit equally well. As Holmes et al. point out, the dynamics of such an evolving and reproducing system is quite complex, there exist no analytical solutions.

We started our experiments with a small bacterial population (10^4^ cells) in order to minimize the chance of having an already resistant mutant in the inoculum. Also, the emergence of a mutant, possessing a single but specific mutation conferring resistance, within 30 generations, by taking into account only the 10^*−*9^/bp/generation spontaneous mutation rate of *E. coli* in stress-free environments^54^, is vanishingly small (see calculation in the Supplementary Information). Still, we saw the emergence of insensitive sub-populations with prolonged phage exposure in each experiment. This suggest that stress-induced mutations induced by the microenvironment also contribute to the observed phenomena. Based on a simple calculation we estimated this mutation rate to be in the order of magnitude 10^*−*5^, which is a vastly higher rate than the mutation rate present under stress-free conditions (supplementary Information). Mutation rates in bacteria can increase by phenotypic stress responses^55^ and genetic mutations^56^, as well. The calculated 10^*−*5^ mutation rate would not be unprecedented given the role of the SOS response and presumable hypoxic conditions in biofilms that we observe can provide in accelerating mutation frequencies^57^. Growth curves of bacteria measured in the presence of T4r phage Fig. 4c suggest that the patchy landscape in the microfluidic device help the selection of resistant populations. No intense growth was observed in the well-mixed liquid co-cultures even at low MOIs.

Phage exposure may alter different phenotypes, including biofilm formation. Growing evidence shows that bacteriophages can modulate biofilm development^58,59^, but most of these studies deal with lysogenic phage and not the virulent ones. The influence of virulent phages on biofilm formation is more limited, and mostly relates to study the eradication of biofilms with high phage titers.

Phage-bacteria interactions could be considerably different in spatially structured contexts as opposed to well-mixed liquid cultures^27–29^. One essential difference is the possibility of cell aggregation and the development of biofilms. Under such circumstances even the evolutionary pathways might differ from those appear in shaken culture test tubes^28^. Besides, the lytic cycle of infected planktonic and anchored cells showed differences due to changes in the nanostructure of the bacterial cell envelope measured by atomic force microscopy^29^.

Our results further strengthen the importance of cell aggregates and biofilm formation in a spatially distributed stress landscape, where bacteria are exposed to phage gradient. We found that in our microfluidic platform, T4r phage exposure alters different phenotypes in *E. coli*, including transient biofilm formation. We observed local aggregation of cells that become the nucleation spots of the fast growing and spreading insensitive sub-populations. In case of putting the mutant back to the same stress environment, this transient cell aggregation is even more pronounced (Fig. 5. and Supplementary Movie 3).

The genetic response of bacteria in our device was heterogeneous and varied from run to run, although there were unifying themes (see Table 1). Stress-induced mutations in genes that affect biofilm formation (*csgB, csgD, rcsC*) together with specific mutations against the adhesion of T4r (*ompC, rfaP*) contribute to the observed resistance. Most of these mutations are well-described in the literature. Phage infection begins with the adsorption of phage to the cell followed by its specific binding to the receptor and DNA injection. In the case of T4, OmpC and some LPS structures are used as receptor. Therefore, mutations observed in these genes could inhibit this first physical interaction between the phage and its host^33,34^. The Rcs phosphorelay system had also proved to contribute to the defense mechanism against phages in bacteria that belong to the family of Enterobacteriaceae^38,60,61^. It is involved in the cellular response to extracytoplasmic stress, and it responds to peptidoglycan damage^62^.

The Rcs signaling system controls the transcription of numerous genes involved in biofilms, e.g., genes that are involved in colanic acid capsule synthesis, production of cell surface-associated structures (flagella, LPS, fimbriae), biofilm formation and cell division^63^. RcsC is an important part of this system^64^. In an extensive study on bacterial genes required by different bacteriophages to infect *E. coli* has been carried out by Rousset et al.^65^. They emphasize the role of the Rcs signaling pathway, since the synthesis of colanic acid capsule gives a shared resistance to phages *λ*, 186, T4. It might act as a physical barrier by masking phage receptors on the cell surface. Mutations in *rcsC*, in general, go with a characteristic mucoid phenotype^38^ that we also observed in the case of mutant 2.

In two mutants we observed changes in *csgB* and *csgD*. These genes are important in biofilm formation, motility, adhesion, curli biosynthesis and influence the expression of stress response genes^66^. Vidakovic et al. used a microfluidic flow chamber and showed that CsgA amyloid fibers not only protect well-grown biofilms but protect cells individually by coating their surfaces and preventing virus attachment. They found similar response against T7 and T5 lytic phages^30^. Based on our results, these genes might be involved in the general defense mechanism against phage infection in structured environments where cell aggregation and/or biofilm formation is allowed.

In our experiments, the CRISPR-Cas system of *E. coli* did not turn on, which is in good agreement with the literature. Efficient protection against phages by CRISPR/CAS-E has not been observed in the non-manipulated wild-type *E. coli* K12 strain. The Cascade (CRISPR-associated complex for antiviral defence) genes form an operon whose expression is repressed under normal laboratory growth by the transcriptional regulator H-NS (histone-like nucleoid structuring protein)^18,19^. It is not known what physiological circumstances are needed to turn it on.

There is a movement now to re-develop phage therapy^67^ as an alternative to antibiotics because of the emergence of “super-bugs” with Multi-antibiotic resistance^68^. Based on our results, in spatially structured environments (e.g., the human body) phage resistant bacterial populations emerge in relatively short time. The patchy landscape can contribute to the selection of mutants with fitness-enhancing adaptation. Therefore, successful therapeutic applications must be carefully designed to account for deep changes in evolution dynamics that can occur in complex environments^41^ and the passage of time to phage exposure^69^.

Our results might help in the successful planning of phage therapy against bacterial infections^70^ since we mimic important aspects of the human body, e.g., heterogeneity, compartmentalization, and the possibility of the development of protective barriers like biofilm. Previously, it has been shown that such structured environment could promote the evolution of resistance against antibiotics^12–15^. Our study shows that rapid emergence of stress-induced phage resistance can also occur in structured ecologies.

## Methods

### Culturing bacteria and phage

Bacteriophage T4r (Carolina Biological Supply Company) and *E. coli* AD62 strain were used in the experiments. AD62 is derived from the K-12 strain AB1157^71^ that has the *λ* -deficiency, which makes it a suitable host for the T4r phage we used in our experiments. The *E. coli* strain was transformed with the pWR21 plasmid^72^ for constitutive green fluorescent protein (eGFP) expression. The bacteriophage concentration was 2 × 10^9^ virus particles/ml in the experiments. The proper phage concentration was measured by standard PFU assay.

Before each experiment bacteria were grown overnight in plastic tubes using 2 ml lysogeny broth (LB) medium supplemented with 100 µg/ml ampicillin at 37 °C in an incubator shaker (200 rpm). Overnight cultures were diluted back in the morning, and cells at a concentration of OD600 = 0.6 (optical density measured at 600 nm) were used for the inoculation of the microfluidic device. The number of N bacteria/ml at OD600 = 0.6 is approximately 2 × 10^8^/ml determined by CFU assay. The volume of the center well of the device is approximately 4 × 10^*−*2^ µl. Thus, we predict and can confirm that we inoculated the center well with approximately *N*_*i*_ = 10^4^ bacteria at *t* = 0.

After the experiments resistant cells were isolated by opening the device and using the silicon part for replica plating on agar plates that were previously coated by 10^9^ particles of T4r^73,74^. The selection plates with the imprints of the device were incubated overnight (16 *h*) at 37 °C. Colonies were picked from the regions of the imprint where resistant growth was observed and further analyzed. The key parameter is the spontaneous rate of single-nucleotide polymorphisms in unstressed *E. coli* Θ_*D*_ ≈ 2.0*×*10^*−*9^/generation^54^.

Optical density measurements (at 600 nm) were carried out to characterize the growth of the wild type *E. coli* strain and the mutants isolated from the experiments. The optical density was measured in 110 µl volumes in 96-well plates by using a BioTek Synergy H1 microplate reader. Overnight cultures (2 ml LB, plastic tubes, 200 rpm, 37 °C) were back-diluted in the morning 500 times. When the optical density of the cultures reached 0.6, 100 µl of the bacterial cultures (with or without dilution) together with 10 µl of bacteriophage solution (with the appropriate phage concentration) were measured into the wells. The duration of the plate reader experiments were 24 h, the well plate was shaken continuously (double orbital, 425 cpm frequency), and the temperature was set to 37 °C. Optical density was measured every 5 min. The surface-adhered biofilm-forming ability of the ancestral and mutant strains was tested using the microtiter plate biofilm assay (based on crystal violet staining)^39^. The assay was performed in a 96-well plate. 100 µl of cell cultures (OD = 0.6, in LB) were loaded in each well and incubated for 48 h at 37 ^*o*^C without phage. Three replicates were made for each sample.

### Microfluidic device setup

We used the basic hexagon array of gradient microhabitats used in ref. ^14^. The microfluidic device was etched in two layers into a silicon wafer. The schematic representation of the device is presented in Fig. 1. It is a network of hexagonal wells that are connected through narrow channels etched to 10 µm depth. The nutrient supply of this network is provided from two side channels that are connected to the outer wells through 100 nm deep nanoslits. The total etched area of the hexagon array is approximately 40 mm^2^.

The top of the etched device is reversibly sealed by a 25 µm thick gas-permeable Lumox membrane (SARSTEDT AG & Co. Nu¨mbrecht, Germany). Sealing of the device top was done by pressurizing the outside of the structure with atmospheric composition air at 2 × 10^4 ^Pa. This sealed the film against the silicon wafer but did not close the 100 nm deep etched nanoslits of the device. However, as we discuss in the text, the finite pressure of the sealing film sometimes allowed bacteria to form highly condensed colonies by pushing the film up.

Medium mixed with phage flow was provided by syringe pump at 5 µl/h throughout the experiments. Before each experiment, the chip was run with LB medium (supplemented with 100 µg/ml ampicillin) and bacteriophage T4r in one of the side channels (always the bottom one in the images) for 20 h to ensure an initial virus gradient and nutrient concentration within the device. The highest phage concentration applied in the side channel was 2 × 10^9^ virus particles/ml. The concentration of bacteriophage within the microfluidic device was calculated for a full 3D model using COMSOL Multiphysics 4.3a software (COMSOL AB, Stockholm, Sweden) over the timescale of the experiment. Within the COMSOL the Transport of Diluted Species physics package was used, in which two concentration were added to simulate the diffusion of the phages from the side channels. From the 3D design the side channels were removed to reduce the complexity of the model. To eliminate the size difference between the nanoslits and the chambers, the mesh was manually adjusted. The diffusion constant of the phage was estimated to be 8 × 10^*−*8^cm^2^/s^75,76^. Bacteria were inoculated into the center of the device with a pipette at 10^4^ cell number into the 0.04 µl volume of the inlet hole. Experiments (three biological replicates) were carried out at 30 °C.

### Image acquisition and analysis

Fluorescence time-lapse microscopy was performed by using a Nikon TE2000-E inverted microscope and a Canon camera (EOS5d). To get a high-resolution image series, in one experiment we used a Nikon 90i upright microscope supplemented with an Andor Neo 5.5 sCMOS camera and a 4X Plan APO *λ* objective. In both setups, a GFP filter set was used to monitor the growth and spatial distribution of bacterial populations within the device. *µ*Manager and NIS Elements software were used to take images every 15 min.

Occupancy data was calculated and used to estimate the biomass of the bacterial population within the device in a similar way as in ref. ^77^. The value of occupancy can vary between 0 and 1. Average occupancy was calculated for each microchamber: a threshold pixel value was defined based off the autofluorescence of the images (after background correction) using FIJI software^78^, and above this value the pixel was considered occupied, otherwise it was vacant. A custom mask was created to use the pixel level data to calculate the average occupancy on microchamber level with the help of a custom Python script. We used the occupancy maps to determine at which positions the insensitive populations emerge within the device.

### Whole genome sequencing

Resistant cells were collected after the experiments on selection plates and clones were further analyzed by whole genome sequencing (The Sequencing Center, Fort Collins, CO 80524, USA). Total DNA was extracted by using the Zymo Research Quick-DNA Fungal/Bacterial Microprep Kit (Catalog No. D6007) according to the manufacturer’s instructions. The Illumina (illumina.com) Nextera XT DNA Library Prep Kit was used to prepare extracted bacteria DNA for sequencing. Bacterial whole genome sequencing was performed on an Illumina MiniSeq short-read sequencer using a standard Illumina workflow and configured for 2 × 150 bp paired-end reads and MiniSeq flow cell.

The sequenced samples included one clone of the ancestral strain and three clones isolated from the experiments. We identified mutations related to T4 phage resistance and examined whether the CRISPR- Cas defense mechanism of *E. coli* -that is mostly repressed in lab cultures^79^—was activated under such circumstances. The raw sequences have been deposited under the study number PRJEB73316 at the European Nucleotide Archive (ENA). Paired-end sequencing with 150 bp read length was used to generate reads. Quality assessment was carried out with FastQ^80^. We trimmed raw reads to remove adapter sequences and PhiX174 contamination using BBduk. Sequence reads were assembled using Unicycler (version v0.4.7)^81^. The assembled sequences were annotated with Prokka (version 1.14.6)^82^. BBMap utilities were applied to obtain assembly metrics’ statistics^83,84^. The annotated genomes were compared to identify the missing genes across the strain and mutants using Roary^85^. We identified SNPs in the mutants by mapping the reads to the wild-type strain using Breseq (version: 0.35.4)^86^.

## Supplementary information

Supplementary Material

A time-lapse fluorescence microscopy recording of a characteristic experiment

time-lapse fluorescence microscopy recording made of the fluorescence snapshots overlaid on top of the calculated concentration profile

A time-lapse fluorescence microscopy recording showing the growth and progression of one of the mutant strains

## Data Availability

The data generated in this study are available from the corresponding author upon reasonable request. The raw sequences are available at the European Nucleotide Archive (ENA) database under the study number PRJEB73316.

## References

[1] Pires, D. P., Melo, L. D. R. & Azeredo, J. Understanding the complex phage-host interactions in biofilm communities. *Annu Rev. Virol.* **8**, 73–94 (2021). 34186004 10.1146/annurev-virology-091919-074222

[2] Carlson, K. & Kozinski, A. W. Nonreplicated DNA and DNA fragments in T4r-bacteriophage particles - phenotypic mixing of a phage protein. *J. Virol.* **13**, 1274–1290 (1974). 4598783 10.1128/jvi.13.6.1274-1290.1974 PMC355447

[3] Burch, L. H., Zhang, L. L., Chao, F. G., Xu, H. & Drake, J. W. The bacteriophage T4 rapid-lysis genes and their mutational proclivities. *J. Bacteriol.* **193**, 3537–3545 (2011). 21571993 10.1128/JB.00138-11 PMC3133318

[4] Wang, X., Liu, Y., Li, K. Y. & Hao, Z. H. Roles of p53-mediated host-virus interaction in coron-avirus infection. *Int. J. Mol. Sci.* **24**, 6371 (2023). 37047343 10.3390/ijms24076371 PMC10094438

[5] Chapelle, V. & Silvestre, F. Population epigenetics: The extent of DNA methylation variation in wild animal populations. *Epigenomes* **6**, 31 (2022). 36278677 10.3390/epigenomes6040031 PMC9589984

[6] Pribis, J. P., Zhai, Y., Hastings, P. J. & Rosenberg, S. M. Stress-induced mutagenesis, gambler cells, and stealth targeting antibiotic-induced evolution. *Mbio* **13**, e0107422 (2022). 35658528 10.1128/mbio.01074-22 PMC9239211

[7] Wielgoss, S. et al. Mutation rate dynamics in a bacterial population reflect tension between adaptation and genetic load. *Proc. Natl. Acad. Sci.* **110**, 222–227 (2013). 23248287 10.1073/pnas.1219574110 PMC3538217

[8] Tenaillon, O., Denamur, E. & Matic, I. Evolutionary significance of stress-induced mutagenesis in bacteria. *Trends Microbiol* **12**, 264–270 (2004). 15165604 10.1016/j.tim.2004.04.002

[9] Moxon, E., Rainey, P. B., Nowak, M. A. & Lenski, R. E. Adaptive evolution of highly mutable loci in pathogenic bacteria. *Curr. Biol.* **4**, 24–33 (1994). 7922307 10.1016/s0960-9822(00)00005-1

[10] Baquero, F. & Negri, M.-C. Challenges: Selective compartments for resistant microorganisms in antibiotic gradients. *BioEssays* **19**, 731–736 (1997). 9264256 10.1002/bies.950190814

[11] Hallatschek, O. Bacteria evolve to go against the grain. *Physics* **5**, 93 (2012).

[12] Greulich, P., Waclaw, B. & Allen, R. J. Mutational pathway determines whether drug gradients accelerate evolution of drug-resistant cells. *Phys. Rev. Lett.* **109**, 088101 (2012). 23002776 10.1103/PhysRevLett.109.088101

[13] Hermsen, R., Deris, J. B. & Hwa, T. On the rapidity of antibiotic resistance evolution facilitated by a concentration gradient. *Proc. Natl. Acad. Sci.* **109**, 10775–10780 (2012). 22711808 10.1073/pnas.1117716109 PMC3390829

[14] Zhang, Q. et al. Acceleration of emergence of bacterial antibiotic resistance in connected microenvi-ronments. *Science* **333**, 1764–1767 (2011). 21940899 10.1126/science.1208747

[15] Nagy, K. et al. Emergence of resistant Escherichia coli mutants in microfluidic on-chip antibiotic gradients. *Front. Microbiol.* **13**, 820738 (2022). 35391738 10.3389/fmicb.2022.820738 PMC8981919

[16] Barrangou, R. et al. CRISPR provides acquired resistance against viruses in prokaryotes. *Science* **315**, 1709–1712 (2007). 17379808 10.1126/science.1138140

[17] Marraffini, L. A. CRISPR-Cas immunity in prokaryotes. *Nature* **526**, 55–61 (2015). 26432244 10.1038/nature15386

[18] Pul, U. et al. Identification and characterization of *E. coli* CRISPR- *cas* promoters and their silencing by H-NS. *Mol. Microbiol.* **75**, 1495–1512 (2010). 20132443 10.1111/j.1365-2958.2010.07073.x

[19] Westra, E. R. et al. H-NS-mediated repression of CRISPR-based immunity in *Escherichia coli* K12 can be relieved by the transcription activator LeuO. *Mol. Microbiol.* **77**, 1380–1393 (2010). 20659289 10.1111/j.1365-2958.2010.07315.x

[20] Pougach, K. et al. Transcription, processing and function of CRISPR cassettes in *Escherichia coli*. *Mol. Microbiol.* **77**, 1367–1379 (2010). 20624226 10.1111/j.1365-2958.2010.07265.x PMC2939963

[21] Mojica, F. J. M. & D´ıez-Villaseñor, C. The on–off switch of CRISPR immunity against phages in. *Escherichia coli. Mol. Microbiol.* **77**, 1341–1345 (2010). 20860086 10.1111/j.1365-2958.2010.07326.x

[22] Díez-Villasenor, C., Almendros, C., García-Martínez, J. & Mojica, F. J. M. Diversity of CRISPR loci in Escherichia coli. *Microbiology* **156**, 1351–1361 (2010). 28206910 10.1099/mic.0.036046-0

[23] Perry, E. B., Barrick, J. E. & Bohannan, B. J. M. The molecular and genetic basis of repeatable coevolution between escherichia coli and bacteriophage T3 in a laboratory microcosm. *Plos One* **10**, e0130639 (2015). 26114300 10.1371/journal.pone.0130639 PMC4482675

[24] Phan, T. V. et al. Bacterial route finding and collective escape in mazes and fractals. *Phys. Rev. X* **10**, 031017 (2020).

[25] Phan, T. V. et al. It doesn’t always pay to be fit: Success landscapes. *J. Biol. Phys.* **47**, 387–400 (2021). 34709534 10.1007/s10867-021-09589-2 PMC8603993

[26] Nagy, K., Ábrahám, Á., Keymer, J. E. & Galajda, P. Application of microfluidics in experimental ecology: The importance of being spatial. *Front. Microbiol.* **9**, 496 (2018). 29616009 10.3389/fmicb.2018.00496 PMC5870036

[27] Attrill, E. L. et al. Individual bacteria in structured environments rely on phenotypic resistance to phage. *PLOS Biology* **19**, e3001406 (2021). 34637438 10.1371/journal.pbio.3001406 PMC8509860

[28] Simmons, E. L. et al. Biofilm structure promotes coexistence of phage-resistant and phage-susceptible bacteria. *mSystems* 5 (ed Bordenstein, S.) 10.1128/mSystems.00877-19. (2020). 10.1128/mSystems.00877-19 PMC7311319 32576653

[29] Abraham, S., Kaufman, Y., Perreault, F., Young, R. & Bar-Zeev, E. Bursting out: linking changes in nanotopography and biomechanical properties of biofilm-forming Escherichia coli to the T4 lytic cycle. *npj Biofilms Microbiomes* **7**, 26 (2021). 33731698 10.1038/s41522-021-00195-7 PMC7969764

[30] Vidakovic, L., Singh, P. K., Hartmann, R., Nadell, C. D. & Drescher, K. Dynamic biofilm architecture confers individual and collective mechanisms of viral protection. *Nat. Microbiol.* **3**, 26–31 (2017). 29085075 10.1038/s41564-017-0050-1 PMC5739289

[31] Phan, T. V. et al. Direct measurement of dynamic attractant gradients reveals breakdown of the Patlak-Keller-Segel chemotaxis model. *Proc. Natl. Acad. Sci. U.S.A.* **121**, e2309251121 (2024). 38194458 10.1073/pnas.2309251121 PMC10801886

[32] Wang, M. Z. et al. Uncovering the determinants of model Escherichia coli strain C600 susceptibility and resistance to lytic T4-like and T7-like phage. *Virus Res* **325**, 199048 (2023). 36681192 10.1016/j.virusres.2023.199048 PMC10194157

[33] Washizaki, A., Yonesaki, T. & Otsuka, Y. Characterization of the interactions between *Escherichia coli* receptors, LPS and OmpC, and bacteriophage T4 long tail fibers. *MicrobiologyOpen* **5**, 1003–1015 (2016). 27273222 10.1002/mbo3.384 PMC5221442

[34] Yu, F. & Mizushima, S. Roles of lipopolysaccharide and outer membrane protein OmpC of Escherichia coli K-12 in the receptor function for bacteriophage T4. *J. Bacteriol.* **151**, 718–722 (1982). 7047495 10.1128/jb.151.2.718-722.1982 PMC220313

[35] Parker, C. T. et al. Role of the rfaG and rfaP genes in determining the lipopolysaccharide core structure and cell surface properties of Escherichia coli K-12. *J. Bacteriol.* **174**, 2525–2538 (1992). 1348243 10.1128/jb.174.8.2525-2538.1992 PMC205891

[36] Louros, N. N., Bolas, G. M. P., Tsiolaki, P. L., Hamodrakas, S. J. & Iconomidou, V. A. Intrinsic aggregation propensity of the CsgB nucleator protein is crucial for curli fiber formation. *J. Struct. Biol.* **195**, 179–189 (2016). 27245712 10.1016/j.jsb.2016.05.012

[37] Sato, T. et al. Role of the inner-membrane histidine kinase RcsC and outer-membrane lipoprotein RcsF in the activation of the Rcs phosphorelay signal transduction system in Escherichia coli. *Microbiol. (Read.)* **163**, 1071–1080 (2017). 10.1099/mic.0.000483 28691662

[38] Wielgoss, S., Bergmiller, T., Bischofberger, A. M. & Hall, A. R. Adaptation to parasites and costs of parasite resistance in mutator and nonmutator bacteria. *Mol. Biol. Evolut.* **33**, 770–782 (2016). 10.1093/molbev/msv270 PMC4760081 26609077

[39] Pseudomonas Methods and Protocols (eds et al. 2014).

[40] Weitz, J. S., Hartman, H. & Levin, S. A. Coevolutionary arms races between bacteria and bacterio-phage. *Proc. Natl. Acad. Sci.* **102**, 9535–9540 (2005). 15976021 10.1073/pnas.0504062102 PMC1172273

[41] Go´mez, P. & Buckling, A. Bacteria-phage antagonistic coevolution in soil. *Science* **332**, 106–109 (2011). 21454789 10.1126/science.1198767

[42] Koskella, B. & Brockhurst, M. A. Bacteria–phage coevolution as a driver of ecological and evolu-tionary processes in microbial communities. *FEMS Microbiol. Rev.* **38**, 916–931 (2014). 24617569 10.1111/1574-6976.12072 PMC4257071

[43] Leclerc, Q. J., Lindsay, J. A. & Knight, G. M. Modelling the synergistic effect of bacteriophage and antibiotics on bacteria: Killers and drivers of resistance evolution. *Plos Comput. Biol.* **18**, e1010746 (2022). 36449520 10.1371/journal.pcbi.1010746 PMC9744316

[44] Mizoguchi, K. et al. Coevolution of bacteriophage PP01 and *Escherichia coli* O157:H7 in continuous culture. *Appl. Environ. Microbiol.* **69**, 170–176 (2003). 12513992 10.1128/AEM.69.1.170-176.2003 PMC152390

[45] Bohannan, B. & Lenski, R. Linking genetic change to community evolution: insights from studies of bacteria and bacteriophage. *Ecol. Lett.* **3**, 362–377 (2000).

[46] Aleklett, K. et al. Build your own soil: exploring microfluidics to create microbial habitat structures. *ISME J* **12**, 312–319 (2018). 29135971 10.1038/ismej.2017.184 PMC5776464

[47] Fijalkowska, I. J., Schaaper, R. M. & Jonczyk, P. DNA replication fidelity in Escherichia coli: A multi-DNA polymerase affair. *Fems Microbiol. Rev.* **36**, 1105–1121 (2012). 22404288 10.1111/j.1574-6976.2012.00338.x PMC3391330

[48] Shee, C., Ponder, R., Gibson, J. L. & Rosenberg, S. M. What limits the efficiency of double-strand break-dependent stress-induced mutation in escherichia coli? *J. Mol. Microbiol. Biotechnol.* **21**, 8–19 (2011). 22248539 10.1159/000335354 PMC3697267

[49] Phan, T. V. et al. Emergence of Escherichia coli critically buckled motile helices under stress. *Proc. Natl. Acad. Sci.* **115**, 12979–12984 (2018). 30498027 10.1073/pnas.1809374115 PMC6304939

[50] Bos, J. et al. Emergence of antibiotic resistance from multinucleated bacterial filaments. *Proc. Natl. Acad. Sci. USA* **112**, 178–183 (2015). 25492931 10.1073/pnas.1420702111 PMC4291622

[51] Koonin, E. V. & Wolf, Y. I. Evolution of microbes and viruses: A paradigm shift in evolutionary biology? *Front. Cellular Infect. Microbiol.* **2**, 119 (2012). 22993722 10.3389/fcimb.2012.00119 PMC3440604

[52] Koonin, E. V. & Wolf, Y. I. Just how Lamarckian is CRISPR-Cas immunity: the continuum of evolvability mechanisms. *Biol. Direct* **11**, s13062 (2016). 10.1186/s13062-016-0111-z PMC4765028 26912144

[53] Holmes, C. M., Ghafari, M., Abbas, A., Saravanan, V. & Nemenman, I. Luria-Delbruck, revisited: The classic experiment does not rule out Lamarckian evolution. *Phys. Biol.* **14**, 055004 (2017). 28825411 10.1088/1478-3975/aa8230

[54] Williams, A. B. Spontaneous mutation rates come into focus in. *DNA Repair* **24**, 73–79 (2014). 10.1016/j.dnarep.2014.09.009 25308085

[55] Foster, P. L. Stress-induced mutagenesis in bacteria. *Crit. Rev. Biochem. Mol. Biol.* **42**, 373–397 (2007). 17917873 10.1080/10409230701648494 PMC2747772

[56] Pal, C., Macia, M. D., Oliver, A., Schachar, I. & Buckling, A. Coevolution with viruses drives the evolution of bacterial mutation rates. *Nature* **450**, 1079–1081 (2007). 18059461 10.1038/nature06350

[57] Pribis, J. P., Zhai, Y., Hastings, P. J. & Rosenberg, S. M. Stress-Induced mutagenesis, gambler Cells, and stealth targeting antibiotic-induced evolution. *mBio* **13**, e0107422 (2022). 35658528 10.1128/mbio.01074-22 PMC9239211

[58] Lourenco, M. et al. The gut environment regulates bacterial gene expression which modulates susceptibility to bacteriophage infection. *Cell Host Microbe* **30**, 556 (2022). 35421351 10.1016/j.chom.2022.03.014

[59] Hosseinidoust, Z., Tufenkji, N. & Van De Ven, T. G. Formation of biofilms under phage predation: considerations concerning a biofilm increase. *Biofouling* **29**, 457–468 (2013). 23597188 10.1080/08927014.2013.779370

[60] Huang, Y.-H., Ferrie’res, L. & Clarke, D. J. The role of the Rcs phosphorelay in Enterobacteriaceae. *Res. Microbiol.* **157**, 206–212 (2006). 16427772 10.1016/j.resmic.2005.11.005

[61] Poranen, M. M. et al. Global changes in cellular gene expression during bacteriophage PRD1 infection. *J. Virol.* **80**, 8081–8088 (2006). 16873264 10.1128/JVI.00065-06 PMC1563795

[62] Laubacher, M. E. & Ades, S. E. The Rcs phosphorelay is a cell envelope stress response activated by peptidoglycan stress and contributes to intrinsic antibiotic resistance. *J. Bacteriol.* **190**, 2065–2074 (2008). 18192383 10.1128/JB.01740-07 PMC2258881

[63] Clarke, D. J. The Rcs phosphorelay: more than just a two-component pathway. *Future Microbiol* **5**, 1173–1184 (2010). 20722597 10.2217/fmb.10.83

[64] Ferriéres, L. & Clarke, D. J. The RcsC sensor kinase is required for normal biofilm formation in Escherichia coli K-12 and controls the expression of a regulon in response to growth on a solid surface: E. coli Rcs regulon. *Mol. Microbiol.* **50**, 1665–1682 (2003). 14651646 10.1046/j.1365-2958.2003.03815.x

[65] Rousset, F. et al. Genome-wide CRISPR-dCas9 screens in E. coli identify essential genes and phage host factors. *PLOS Genetics* **14** (ed Blokesch, M.) e1007749 (2018). 10.1371/journal.pgen.1007749 PMC6242692 30403660

[66] Yan, C.-H. et al. The transcription factor CsgD contributes to engineered Escherichia coli resistance by regulating biofilm formation and stress responses. *Int. J. Mol. Sci.* **24**, 13681 (2023). 37761984 10.3390/ijms241813681 PMC10530992

[67] Chanishvili, N. Phage Therapy-History from Twort and d’Herelle Through Soviet Experience to Current Approaches. *Adv. Virus Res., Vol. 83: Bacteriophages, Pt B* **83**, 3–40 (2012). 10.1016/B978-0-12-394438-2.00001-3 22748807

[68] Turner, P. E. et al. Addressing the research and development gaps in modern phage therapy. *Phage-Ther. Appl. Res.* **5**, 30–39 (2024). 10.1089/phage.2023.0045 PMC11920706 40114805

[69] Koderi Valappil, S. et al. Survival comes at a cost: A coevolution of phage and its host leads to phage resistance and antibiotic sensitivity of Pseudomonas aeruginosa multidrug resistant strains. *Front. Microbiol.* **12**, 783722 (2021). 34925289 10.3389/fmicb.2021.783722 PMC8678094

[70] Diallo, K. & Dublanchet, A. Benefits of combined phage-antibiotic therapy for the control of antibiotic-resistant bacteria: A literature review. *Antibiotics-Basel* **11**, 839 (2022). 35884092 10.3390/antibiotics11070839 PMC9311689

[71] DeWitt, S. K. & Adelberg, E. A. The occurence of a genetic transposition in a strain of E. coli. *Genetics* **47**, 577–585 (1962). 17248104 10.1093/genetics/47.5.577 PMC1210353

[72] Pilizota, T. & Shaevitz, J. W. Fast, Multiphase volume adaptation to hyperosmotic shock by escherichia coli. *PLoS ONE* **7**, e35205 (2012). 22514721 10.1371/journal.pone.0035205 PMC3325977

[73] Luria, S. E. & Delbruck, M. Mutations of bacteria from virus sensitivity to virus resistance. *Genetics* **28**, 491–511 (1943). 17247100 10.1093/genetics/28.6.491 PMC1209226

[74] Lederberg, J. & Lederberg, E. M. Replica plating and indirect selection of bacterial mutants. *J. Bacteriol.* **63**, 399–406 (1952). 14927572 10.1128/jb.63.3.399-406.1952 PMC169282

[75] Polson, A. & Shepard, C. On the diffusion rates of bacteriophages. *Biochimica et. Biophysica Acta* **3**, 137–145 (1949).

[76] Ghaeli, I., Hosseinidoust, Z., Zolfagharnasab, H. & Jorge Monteiro, F. A new label-free technique for analysing evaporation induced self-assembly of viral nanoparticles based on enhanced dark-field optical imaging. *Nanomaterials* **8**, 1 (2017). 29271875 10.3390/nano8010001 PMC5791088

[77] Wetherington, M. T. et al. Ecological succession and the competition-colonization trade-off in microbial communities. *BMC Biol* **20**, 262 (2022). 36447225 10.1186/s12915-022-01462-5 PMC9710175

[78] Schindelin, J. et al. Fiji: An open-source platform for biological-image analysis. *Nat. Methods* **9**, 676–682 (2012). Publisher: Nature Publishing Group. 22743772 10.1038/nmeth.2019 PMC3855844

[79] Yosef, I., Goren, M. G., Kiro, R., Edgar, R. & Qimron, U. High-temperature protein G is essen- tial for activity of the Escherichia coli clustered regularly interspaced short palindromic repeats (CRISPR)/Cas system. *Proc. Natl. Acad. Sci. USA* **108**, 20136–20141 (2011). 22114197 10.1073/pnas.1113519108 PMC3250196

[80] Wingett, S. W. & Andrews, S. FastQ screen: A tool for multi-genome mapping and quality control. *F1000Research* **7**, 1338 (2018). 30254741 10.12688/f1000research.15931.1 PMC6124377

[81] Wick, R. R., Judd, L. M., Gorrie, C. L. & Holt, K. E. Unicycler: Resolving bacterial genome assemblies from short and long sequencing reads. *PLOS Comput. Biol*. **13** (ed Phillippy, A. M.) e1005595 (2017). 10.1371/journal.pcbi.1005595 PMC5481147 28594827

[82] Seemann, T. Prokka: rapid prokaryotic genome annotation. *Bioinformatics* **30**, 2068–2069 (2014). 24642063 10.1093/bioinformatics/btu153

[83] Lundgren, A. & Kanewala, U. Experiences of Testing Bioinformatics Programs for Detecting Subtle Faults. *Proceedings of 2016 Ieee/Acm International Workshop on Software Engineering for Science (Se4science)*, 16–22 (2016).

[84] Bushnell, B., Rood, J. & Singer, E. BBMerge - Accurate paired shotgun read merging via overlap. *Plos One* **12**, e0185056 (2017). 29073143 10.1371/journal.pone.0185056 PMC5657622

[85] Page, A. J. et al. Roary: rapid large-scale prokaryote pan genome analysis. *Bioinformatics* **31**, 3691–3693 (2015). 26198102 10.1093/bioinformatics/btv421 PMC4817141

[86] Deatherage, D. E. & Barrick, J. E. In *Engineering and Analyzing Multicellular Systems* (eds Sun, L. & Shou, W.) Series Title: Methods in Molecular Biology, 165–188 (Springer New York, New York, NY, 2014).

