# Supplementary material for: Gradient metapopulation microfluidic ecologies shape genetic and biofilm drivers of T4r phage resistance in E. coli
Source: NPJ Biofilms Microbiomes. 2026 Apr 25;12:129. doi: 10.1038/s41522-026-00959-z (PMC13324545; doi:10.1038/s41522-026-00959-z)
Supplement: Supplementary file 1 — Supplementary Material [file 41522_2026_959_MOESM1_ESM.pdf]

Supplemetary information for

Gradient metapopulation microfluidic ecologies shape genetic and biofilm drivers of T4r phage resistance in *E. coli*

Krisztina Nagy[1,2], Sarshad Koderi Valappil[3,4], Trung V. Phan[5], Shengkai Li[6], Laszlo Der[1], Ryan Morris[7], Julia Boss[8], Sophia Winslow[9], Peter Galajda[1], Gabor Rakhely[1,3], Robert H. Austin[6*]

[1]*Institute of Biophysics, HUN-REN Biological Research Centre, Szeged, Hungary*
[2]*Department of Experimental Physics, Institute of Physics, University of Szeged, Szeged, Hungary*
[3]*Department of Biotechnology, University of Szeged, Szeged, Hungary*
[4]*Environmental Genomics and Systems Biology Division, Lawrence Berkeley National Laboratory, Berkeley, CA, US*
[5]*Department of Chemical and Biomolecular Engineering, Johns Hopkins University, Baltimore, MD, USA*
[6]*Department of Physics, Princeton University, Princeton, NJ, USA*
[7]*School of Physics & Astronomy, University of Edinburgh, Edinburgh, UK*
[8]*Institut Pasteur, Université Paris Cité, CNRS UMR 3525, Unité Plasticité du Génome Bactérien, Paris, France*
[9]*University of Northwestern St. Paul, Roseville, MN, USA*

[*]*Correspondence*: austin@princeton.edu

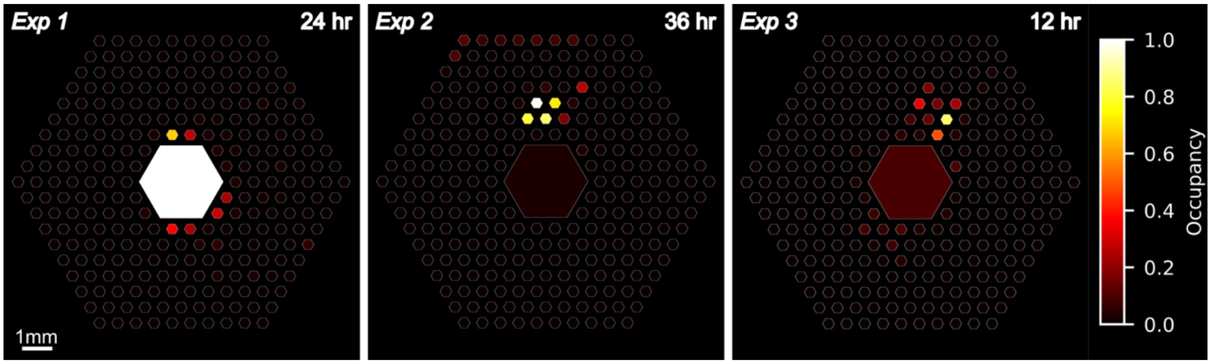

Supplementary Figure 1: Occupancy map of the three independent experiments at the timepoint when the insensitive bacteria population start intense growth. Images show that the ”hot-spots” are located in the regions with low/intermediate phage concentrations. The average occupancy of the microchambers is color-coded.

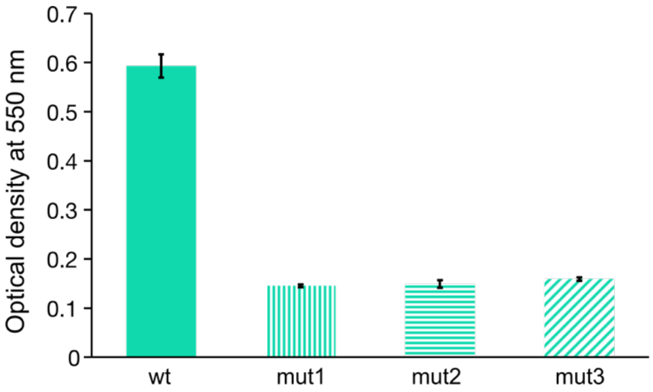

Supplementary Figure 2: Biofilm forming ability of the ancestral and the mutant strains evolved in the microfluidic device in the presence of bacteriophage T4r gradient. The applied crystal violet staining shows the amount of surface-adhered biofilm after 48 hours of incubation at 37°C. Three replicates were performed for each sample.

Estimation on mutation rates in the microfluidic setup

We can make some simple assumptions to guess how many pre-existing mutants were in the original inoculation. In a "worst case" scenario, assume that a single but specific basepair mutation suffices to give phage resistance. If we start with N_o bacteria and end with N bacteria, the total number of bacteria that ever lived is $2N - N_o$. In the absence of stress (no phage contact) the spontaneous mutation rate is $\Theta_D \sim 10^{-9}$ basepairs/generation. This means that for a single resistant mutation (as opposed to any mutation), in the first generation $\Theta_D N_o$ are resistant, and $N_o(2 - \Theta_D)$ are sensitive. After a total of G generations we will have $[N_o(2 - \Theta_D)]2^{G-1}$ sensitive mutants, and $(\Theta_D N_o)2^{G-1}$ resistant ones. It is possible to then continue down this line from generation to generation, in each generation of $N_o 2^{G-1}$ total bacteria we again get $\theta_D N_o 2^{G-1}$ resistant mutants, assuming that the pool of sensitive bacteria has not changed greatly due to $\Theta_D << 1$. Under the assumption of a relatively unchanged pool for mutants from generation to generation, we sum to get the total number of resistant mutants N_r starting with N_o sensitive mutants in G generations:

$$N_r \sim G\Theta_D N_o 2^{G-1}$$

This yields for the fraction $\mathcal{F}$ of bacteria that have resistance due to purely spontaneous mutations Θ_D after G generations:

$$\mathcal{F} \sim \frac{G\Theta_D N_o 2^{G-1}}{N_o 2^{G-1}} = G\Theta_D$$

Thus, only $G\Theta_D N_o$ bacteria would have been expected to be already resistant due to spontaneous mutations. In our case $G \sim 30$ for the initial expansion, the number of expected already resistant bacteria in the initial inoculation of $N_o \sim 10^4$ bacteria is vanishingly small, while we saw resistance emerge with prolonged phage exporure in each experiment.

We can attempt a very rough estimate of the stress-induced mutation rate Θ_L in a similar way, assuming that only 1 mutation can give rise to resistance. We calculate with 15 hrs of incubation (roughly 30 generations), starting with $N_o \sim 10^4$. Assuming we see at least 1 resistant mutant in 30 generations, this yields the mutation rate Θ_L:

$$\Theta_L \sim \frac{1}{30 \times 10^4} \sim 10^{-5}$$

This is a vastly higher rate than the spontaneous mutation rate Θ_D present under stress-free conditions. However, it is not unprecedented given the role the SOS response and presumable hypoxic conditions in biofilms that we observe can provide in accelerating mutation frequencies.

Captions for Supplementary Movies

Supplementary Movie 1: A time-lapse fluorescence microscopy recording of a characteristic experiment (75 hour long). The growth and progression of *E. coli* in the presence of bacteriophage T4r gradient can be seen in the video.

Supplementary Movie 2: A time-lapse fluorescence microscopy recording made of the fluorescence snapshots overlaid on top of the calculated concentration profile over the 75-hour-long experiment (the same experiment as in Supplementary Movie 1).

Supplementary Movie 3: A time-lapse fluorescence microscopy recording showing the growth and progression of one of the mutant strains in the presence of bacteriophage T4r gradient. During the 20 hours long video we can observe the chemotactic waves typical for *E. coli* and the transient aggregation of bacteria in the high phage concentration side of the microfluidic device.
